# Computational physiological models for individualised mechanical ventilation: a systematic literature review focussing on quality, availability, and clinical readiness

**DOI:** 10.1186/s13054-023-04549-9

**Published:** 2023-07-06

**Authors:** R. S. P. Warnaar, M. P. Mulder, L. Fresiello, A. D. Cornet, L. M. A. Heunks, D. W. Donker, E. Oppersma

**Affiliations:** 1 Cardiovascular and Respiratory Physiology, Technical Medical Centre, University of Twente, P.O. Box 217, 7500 AE Enschede, The Netherlands; 2 Department of Intensive Care, Medisch Spectrum Twente, Enschede, The Netherlands; 3 Department of Intensive Care, Erasmus University Medical Centre, Rotterdam, The Netherlands; 4 Intensive Care Centre, University Medical Centre Utrecht, Utrecht, The Netherlands

**Keywords:** Computational physiological model, Physiological modelling, Mechanical ventilation, Respiratory failure, Systematic review, Decision support

## Abstract

**Background:**

Individualised optimisation of mechanical ventilation (MV) remains cumbersome in modern intensive care medicine. Computerised, model-based support systems could help in tailoring MV settings to the complex interactions between MV and the individual patient's pathophysiology. Therefore, we critically appraised the current literature on computational physiological models (CPMs) for individualised MV in the ICU with a focus on quality, availability, and clinical readiness.

**Methods:**

A systematic literature search was conducted on 13 February 2023 in MEDLINE ALL, Embase, Scopus and Web of Science to identify original research articles describing CPMs for individualised MV in the ICU. The modelled physiological phenomena, clinical applications, and level of readiness were extracted. The quality of model design reporting and validation was assessed based on American Society of Mechanical Engineers (ASME) standards.

**Results:**

Out of 6,333 unique publications, 149 publications were included. CPMs emerged since the 1970s with increasing levels of readiness. A total of 131 articles (88%) modelled lung mechanics, mainly for lung-protective ventilation. Gas exchange (*n* = 38, 26%) and gas homeostasis (*n* = 36, 24%) models had mainly applications in controlling oxygenation and ventilation. Respiratory muscle function models for diaphragm-protective ventilation emerged recently (*n* = 3, 2%). Three randomised controlled trials were initiated, applying the Beacon and CURE Soft models for gas exchange and PEEP optimisation. Overall, model design and quality were reported unsatisfactory in 93% and 21% of the articles, respectively.

**Conclusion:**

CPMs are advancing towards clinical application as an explainable tool to optimise individualised MV. To promote clinical application, dedicated standards for quality assessment and model reporting are essential.

*Trial registration number* PROSPERO—CRD42022301715. Registered 05 February, 2022.

**Supplementary Information:**

The online version contains supplementary material available at 10.1186/s13054-023-04549-9.

## Introduction

Mechanical ventilation (MV) is a mainstay of critical care support in acute and severe respiratory failure. Despite its widespread use in modern intensive care medicine, individualised MV optimisation remains cumbersome [1] and time-consuming [2]. This is largely due to limited monitoring of how the mechanical ventilator interacts with the diseased lung in individual patients at bedside. The complexity of continuously aiming for optimal MV settings is further increased by patient heterogeneity and the dynamicity of clinical courses [2]. Moreover, multiple therapeutic targets, i.e. gas exchange but also lung- and diaphragm-protective ventilation, require specific and at times conflicting MV settings [2]. The limited mechanistic insight in the dynamic patient condition may cumulate in suboptimally tailored MV settings, which are associated with increased mortality [3].

**Fig. 1:**
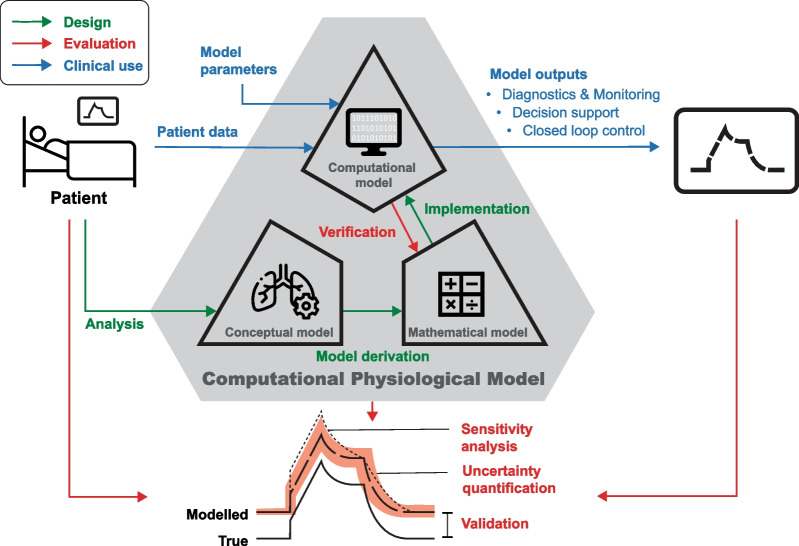
Design, evaluation and clinical use of computational physiological models—Model design (green) requires analysis of the essential physiological concepts, derivation of its mathematical model, and computer implementation. Evaluation (red) comprises validation, uncertainty quantification, and sensitivity analysis, i.e. the susceptibility of the model outcomes to variations in input data and model parameters. During verification, the outcomes of computer implementation are compared to known analytical solutions of the mathematical model. For clinical use (blue), patient data and the (non-individualised) model parameters serve as input for the computer model, which outputs clinically useful information

To optimise MV settings to the individual patient condition, computerised support systems have been developed since the 1980s, with applications in diagnostics, monitoring, decision support, and closed loop control of MV (Fig. 1) [4]. These systems started out as rule-based, rigid implementations of clinical protocols [1, 4], of which SmartCare®/PS, (Drägerwerk AG & Co. KGaA, Lübeck, Germany) is an advanced example. In the pursuit of robust and widely applicable techniques to facilitate individualised MV, recent advances in computational technology have stimulated the emergence of data-driven computerised decision support. This has fostered the use of artificial intelligence (AI) to analyse vast amounts of patient data in intensive care medicine including MV [5]. Yet, these data-driven approaches are usually based on clinical cohorts and cannot account for the detailed pathophysiology of a unique patient [6, 7]. This inherent shortcoming of AI can likely be overcome by model-based systems that incorporate all relevant pathophysiological elements of an individual patient [8]. Such ‘first principles-based (e.g. physics-based or mechanistic) computational models’ are defined by the Food and Drug Administration (FDA) as computational physiological models (CPM) [9]. CPMs may act as virtual patients, allowing to tailor MV settings and assess their effects based on the individual patient's pathophysiology [1, 8].

From a clinical perspective, the CPM concept is strongly aligned with the common daily practice of pathophysiological reasoning at the bedside of intensive care patients. In this sense, CPMs for MV may better serve the important clinical requirements of decision support systems as explainability and credibility, both considered inherent limitations of AI. Recently, the potential of CPMs has also caught attention of leading regulatory bodies, investing significantly to facilitate regulatory frameworks [9, 10]. Therefore, we set out to systematically appraise the current literature on CPMs for individualised MV management in intensive care medicine with a focus on availability, quality, and clinical readiness.

## Methods

This review has been designed in accordance with the Preferred Reporting Items for Systematic reviews and Meta-analysis (PRISMA) guideline with a checklist displayed in Additional file 1 and was registered in the PROSPERO database before initiation of the literature search (PROSPERO 2022 CRD42022301715).

### Study identification and selection

A comprehensive search was performed using MeSH terms and free text variations of the core concepts ‘computational models’ of ‘respiratory physiology’ during ‘mechanical ventilation’ and applicability in ‘individualised’ MV. The MEDLINE ALL, Embase, Scopus and Web of Science literature databases were systematically searched from database creation until 13 February 2023. The full search queries can be found in Additional file 2. Original research articles were eligible for inclusion if they discuss a CPM of the respiratory system that was applicable for individualised MV management, i.e. diagnosis, monitoring or setting, in individual adult ICU patients. For a model described in an article to be marked as a CPM, the expression and computation of at least one physiological quantity are required in relation to one or more other physiological quantities. Articles were excluded if the full text was not available, or if it was not written in or translated to English. The reference lists of eligible studies were searched for additional articles meeting the screening criteria.

Both the screening of title and abstract and the full-text screening were performed in Rayyan by two researchers (RW and MdB). Identical abstracts were removed automatically using Mendeley (Mendeley Desktop, Version 1.19.9, Mendeley Ltd.). Remaining duplicates were removed in Rayyan (Rayyan Systems, Inc., Cambridge, MA, USA). Disagreements were resolved by a third researcher (EO).

### Data extraction

Model characteristics were extracted from the included articles using a predefined online extraction form in the platform SRDR + (AHRQ & Brown University, RI, USA) (Additional file 3). Data were extracted regarding the modelled physiological phenomena in relation to the clinical application, the model maturity, and the model availability.

The modelled physiological phenomena describe which concepts of lung mechanics, gas exchange, gas homeostasis, respiratory control, and respiratory muscle function were included in the model (Table 1). Clinical applications were categorised by diagnosing or monitoring physiological characteristics, advising on ventilator settings, or other.

**Table 1:** Definitions of physiological phenomena

Physiological phenomenon	Describes relations between …
Lung mechanics	… pressures, flows and/or volumes in relation to compliance(s) and/or resistance(s)
Gas exchange	… alveolar, venous and/or arterial partial gas pressures in relation to dead space, shunt, and/or diffusion
Gas homeostasis	… gas contents of blood and/or other bodily compartments
Respiratory control	… the measured physiological control variables (e.g. pH, PaCO_2_, PaO_2_) and the required respiratory response (e.g. respiratory rate, tidal volume, minute ventilation)
Respiratory muscle function	… neural activation and mechanical output of respiratory muscles

Model maturity was graded per article according to its clinical level of readiness. These were previously translated from the general technology level of readiness of the National Aeronautics and Space Administration (NASA) to a clinical level of readiness [11]. The clinical level of readiness indicates the maturation process of a clinical technology from its conceptualisation (level 1) up to post market research (level 9).

Model availability was assessed by publication of the mathematical model and the model parameters, and availability of the computational model (Fig. 1). If the computer model was available, it could be the open-source code, a free software application, or a commercialised software application, either as a stand-alone application or incorporated in a medical device.

### Model quality

The model quality was assessed in accordance with the American Society of Mechanical Engineers (ASME) Verification & Validation (V&V) 40–2018 Standard for Assessing the credibility of computational models [12], in terms of model characterisation and model validation. Classification scales for model quality (see Additional file 4) resulted from in-depth consensus discussions with multiple authors (RW, MM, EM, LF).

Model characterisation was assessed on the comprehensive description of the CPM design and evaluation process (model assumptions, sensitivity analysis, input data uncertainty quantification, and model verification, Fig. 1). These items were scored as unknown, none, partial or full.

Model validation was assessed on the validation sample, the rigor of output comparison, and the agreement of output comparison. These three items were scored as unsatisfactory, partly satisfactory, or satisfactory, if model validation was performed within the specific article. In case information was missing in the main article, supplementary materials were checked for the missing information. If any model characteristics remained unclear, they were reported as ‘unknown’.

### Data analysis

Modelled physiological phenomena and clinical applications were tabulated and aggregated in pivot tables. Model maturity was plotted as publication count per level of readiness against publication year. Model availability was calculated as the proportion of articles of which the mathematical model, computational model, and model parameters were available. Quality assessment criteria were calculated as proportion of articles attaining a specific score per item. Overall scores for model characterisation and validation were calculated as the percentage of articles attaining at least that specific score on all items for model characterisation and validation, respectively.

## Results

### Study identification

**Fig. 2:**
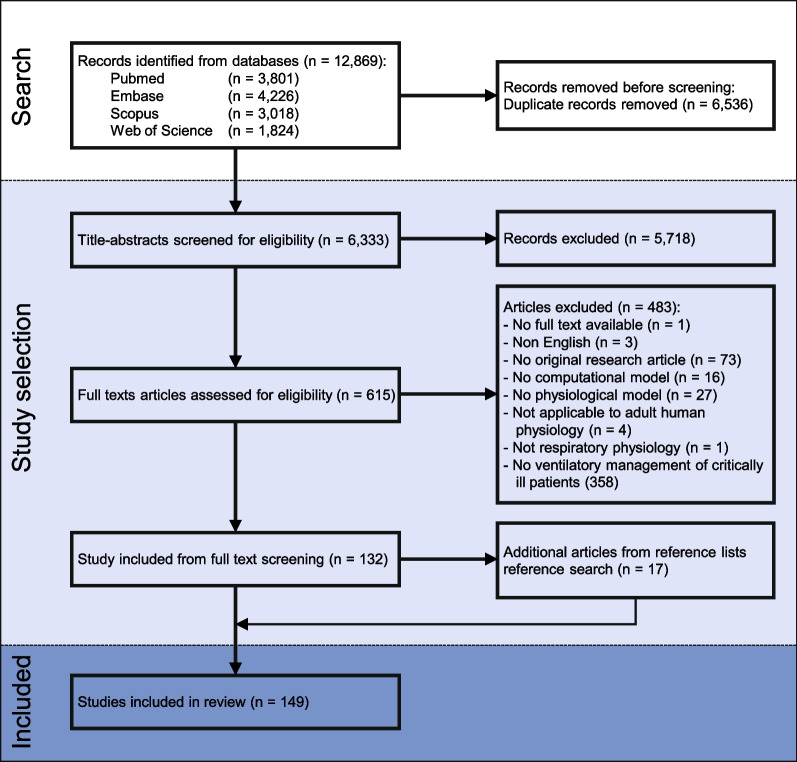
Study selection flow diagram

A total of 6,333 unique publications were identified, of which 5,718 and 483 publications were excluded during the title-and-abstract and full-text screening, respectively, yielding 132 eligible articles (Fig. 2). Seventeen additional articles were identified during the reference screening, resulting into a total of 149 included articles. The main reason for exclusion (*n* = 358) during the full-text screening was that the CPM was not applicable for MV setting in individual critically ill patients (Fig. 2). A list of these excluded articles can be found in Additional file 5.

### Clinical applications

Most articles (*n* = 104, 70%) modelled lung mechanics as single physiological phenomenon (Table 2). These models were all intended to optimise lung mechanics, either directly by advising on optimal PEEP, driving pressure or tidal volume, or indirectly by characterising the patient’s lung mechanics (Additional file 6). Three articles modelled respiratory muscle function. Six articles included all other four predefined phenomena (Table 1): lung mechanics, gas exchange, gas homeostasis, and respiratory control. Articles including three phenomena (*n* = 20) did not include respiratory control in 13 cases. In 40 (27%) articles, two or more physiological phenomena were modelled, of which the combination of gas exchange and gas homeostasis was most prevalent (*n* = 35). Commonly, the clinical application when modelling three or four physiological phenomena (*n* = 26) was to control oxygenation (*n* = 21), by advising on PEEP or FiO_2_, or to control CO_2_ elimination (*n* = 24), by advising on respiratory rate and driving pressure or tidal volume. An overview of all modelled phenomena and clinical applications per article is available in Additional file 6.

**Table 2:**
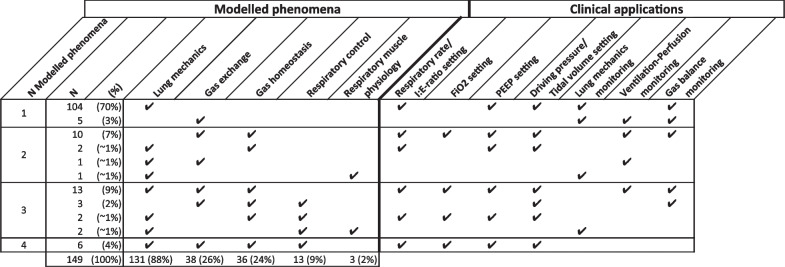
Modelled phenomena combinations and their clinical applications

### Model maturity

**Fig. 3:**
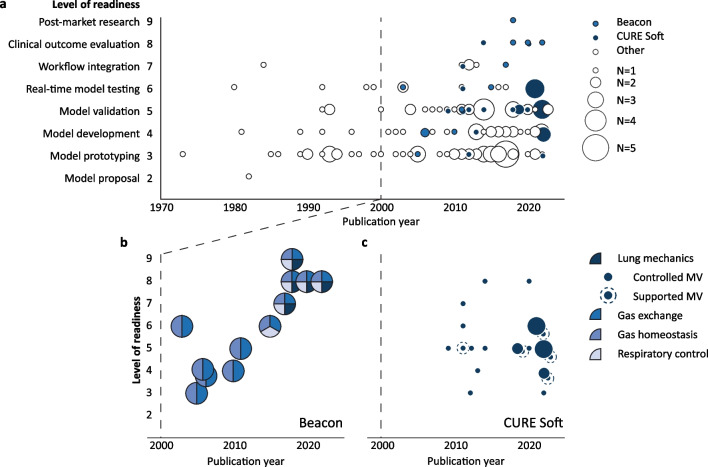
Publication counts and level of readiness over time—**a** Publication counts and level of readiness over time of all included models. **b, c** The included physiological phenomena over time are shown for the models with a level of readiness of eight or higher—Beacon (**b**) and CURE soft (**c**). Marker size indicates the number of articles

CPMs are emerging in clinical research, as reflected by both the increasing publication counts and level of readiness over time (Fig. 3a). Ten out of 13 articles including respiratory control, 3 out of 3 articles including respiratory muscle physiology, and 5 out of 6 articles including four physiological phenomena were published over the last decade alone.

The publication of randomised controlled trial (RCT) protocols (*n* = 3) on the Beacon (trial registration number NCT03249623 [13] and NCT04115709 [14]) and CURE Soft models (trial registration number ACTRN12614001069640 [15]) mark the advent of CPMs reaching the level of clinical outcome evaluation (level 8 or 9). The development of these models is shown in detail in Fig. 3b, c. Beacon started as a model of two physiological phenomena (gas exchange and homeostasis) and was expanded by adding physiological phenomena (Fig. 3b). Articles by the CURE Soft group modelled lung mechanics, differentiating between controlled and supported modes of MV (Fig. 3c).

### Model availability

Nearly all articles (*n* = 147, 99%) indicated their mathematical model was publicly available, and 97 (65%) articles presented the model parameters. A total of 12 (8%) articles published the computer model itself, of which 2 (1%) as open-source code, 2 (1%) as a free and 8 (5%) as a commercialised software application.

### Model quality

**Fig. 4:**
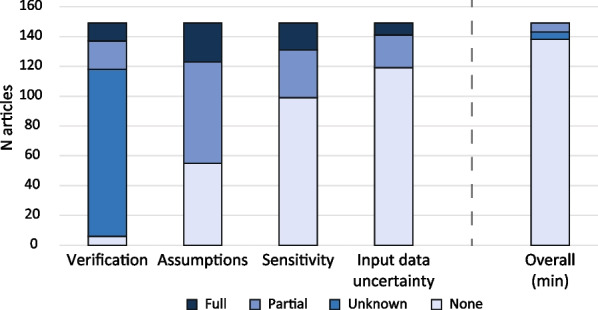
Model characteristics—the overall column indicates the minimal score an article obtained over all studied model characteristics

Most articles described their model assumptions (Fig. 4), either partially (*n* = 68, 46%) or fully (*n* = 26, 17%). Model sensitivity analysis (*n* = 99, 66%) and input data uncertainty quantification (*n* = 119, 80%) were often not described. None of the articles fully described all quality characteristics (Fig. 1), and only 6 (4%) articles discussed all quality characteristics either partially or fully. Information regarding model verification was often missing (*n* = 112, 75%) and hence reported as unknown.

**Fig. 5:**
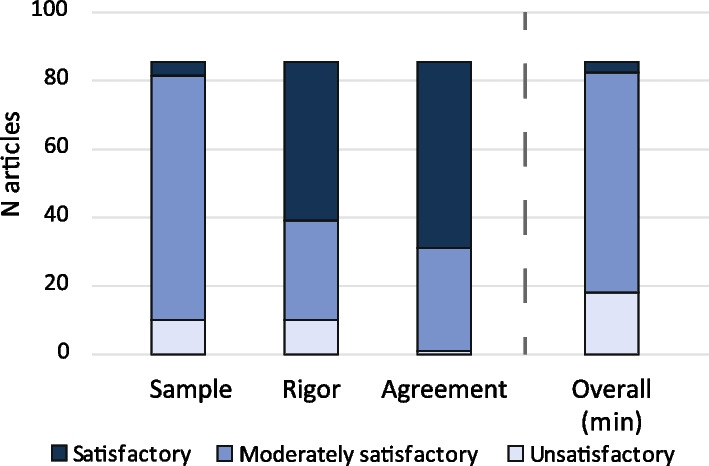
Model validation—the overall column indicates the minimal score an article obtained over all model validation criteria. Only the articles that performed some validation (*n* = 85) are included

The quality of validation processes is shown in Fig. 5. Most of the articles that did some form of validation (*n* = 85) performed partly satisfactory (*n* = 64, 75%) to satisfactory (*n* = 3, 4%). The validation sample was partly satisfactory in 71 (84%) articles. Of those articles that did not perform any validation (*n* = 64), 15 reported prior model validation.

## Discussion

This review systematically appraised the clinical readiness, availability, and quality of CPMs for individualised MV management in intensive care medicine. Publication counts on these models increase since the 1970s with a focus on lung mechanics, while recently a progressive rise is noted covering all relevant topics in modern MV management. Two models are approaching implementation in routine clinical practice, as they are embedded in clinical outcome evaluations through randomised controlled trials [13–15]. Model design and quality characteristics were overall reported insufficiently. Standardisation and transparency of model quality assessment could promote re-use, further development, and more widespread and safe clinical application of CPMs for individualised MV in daily practice.

### Advance of CPMs for individualised MV

The main clinical challenges in individualised MV centre around lung- and diaphragm-protective ventilatory strategies [2]. Lung-protective ventilation involves optimisation of ventilatory pressures and volumes [3, 16] accounting for lung mechanics while maintaining adequate gas exchange. The majority of studies on CPMs for lung-protective ventilation however merely modelled lung mechanics as a single physiological phenomenon, whereas models including gas exchange and gas homeostasis were less prevalent. This might render most of these models incomprehensive from a clinical perspective. Regarding CPMs dedicated to diaphragm-protective ventilation, which has caught increasing clinical attention in recent years, only three articles modelled respiratory muscle function so far [17–19], and an additional four studies were able to quantify work of breathing using lung mechanics models [20–23]. Four articles used CPMs for the detection of patient-ventilator asynchronies [24–27], which is relevant to both lung- and diaphragm-protective ventilation strategies [2]. Overall, literature on CPMs for individualised MV was primarily focussed on lung-protective ventilation, while gas exchange and diaphragm-protective ventilation were underrepresented.

The most important explanation for this underrepresentation of these models is the still incomplete understanding of the pathophysiology of diaphragmatic dysfunction in relation to MV settings. Moreover, the required model complexity in this intricate context, and the large amount of patient data required for adequate CPM design contribute to the underrepresentation of these models.

CPM development necessitates thorough understanding of the pathophysiological concepts underlying the challenges of individualised MV. Modelling these concepts with the right degree of complexity while still being personalisable at the clinical bedside is the subsequent challenge. CPMs are well documented and accepted for describing lung mechanics, as also reflected by their extensive use in fundamental pathophysiological research since the 1950s [28, 29]. Although models of gas homeostasis have likewise been established since the 1970s [30], gas homeostasis interacts closely with other bodily functions, such as the cardiac function, metabolic demands, renal function, and the intra- and extravascular fluid balances. Achieving representative model complexity while still being personalisable at the clinical bedside might therefore be more challenging for gas homeostasis compared to lung mechanics models, possibly explaining the predominance of the latter. The sequelae of MV for respiratory muscle function, on the other hand, had not been established until the 2000s [31]. The physiological targets for diaphragm-protective ventilation are still subject of an ongoing debate [2]. Without relevant physiological targets, CPMs are less powerful when aiming for individualised MV [6], possibly explaining the current dearth of respiratory muscle function models, as shown in this review. Similarly, the recent advances regarding patient-ventilator asynchronies might have sparked the development of models for detecting asynchronies. The availability and processing of relevant patient data has so far been another important aspect that may have hampered CPM development. Using readily available (patho-)physiological patient data is an efficient way to develop CPMs towards clinical applicability (Fig. 1). The data required to personalise many lung mechanics models in this review, i.e. ventilator pressures and flows, have been readily available at bedside since the introduction of mechanical ventilation, yet continuous clinical data acquisition is approaching a stage of practical usability. In practice, suitable data for gas exchange and gas homeostasis models, e.g. continuous capnography and pulse-oximetry, have been routinely available in clinical practice since the 1980s. On the other hand, innovative technologies that find a more widespread clinical use, such as electrical impedance tomography, respiratory muscle monitoring using esophageal pressure catheters [32] and electromyography, are still reserved to a limited number of patients and ICUs, while such data are essential for developing respiratory muscle function models. Acquiring new data that captures yet uncovered aspects of the physiology could spark CPM development for more individualised MV. However, adoption of new measuring modalities often requires laborious changes in the clinical workflow and can hence also complicate model development [6].

So far, the Beacon [13, 14] and CURE Soft [15] CPMs are in the process of clinical outcome evaluation. Beacon aims for both lung-protective ventilation and work of breathing optimisation, where CURE Soft advices on PEEP optimisation. The success of these models might have been enabled by aiming for clinical utility from the start of model conception. Model complexity of both the Beacon [30] and CURE Soft [33] models was aligned with the clinical context of use, while still being bedside personalisable, i.e. a correct degree of complexity. Moreover, both models developed gradually over time (Fig. 3). They first demonstrated a proof of concept in studies with a lower clinical level of readiness, e.g. in silico or in vivo with animal or healthy human subjects (level 3–4). Thereafter, the model was validated (level 5), culminating in patient studies showing its clinical utility (level 6–9). Currently, the Beacon model is commercialised as the ‘Beacon Caresystem’ (Mermaid Care A/S, Sundby, Denmark) and as such CE marked, FDA cleared, and ISO certified as medical device [34].

### CPMs and artificial intelligence

This review showed that the clinical applications of CPMs are conceptually closely aligned with the patient pathophysiology they aim to simulate (Table 2), providing recommendations for MV optimisation based on mechanistic insights. With the advance of innovative digital technology in critical care, both physiological and AI models have been introduced as promising solutions for individualised MV [7, 8]. The underlying technological concepts differ however fundamentally, raising the question how these solutions relate to each other in pursuing further optimisation of individualised MV. The applications of CPMs in this review differ markedly from those previously reported for AI in MV [7]. AI methods are generally employed for forecasting more abstract clinical phenomena, such as predicting weaning success, MV commencement, and MV complications [7]. Only four AI models are aimed at clinical decision support for MV settings [7], of which three were trained to reproduce expert clinical decision-making [35, 36]. Reproduction of expert clinical decision-making might suffice for supporting innovation to optimise MV, but it does not provide mechanistic insights into the individual patient management.

Although CPMs and AI have generally distinct applications in MV management, these conceptually different methodological strategies may strongly be complementary. As an integrative example, one of the AI based MV decision support systems reported above, uses an AI approach to estimate some of the CPM parameters from patient data [37]. The resultant CPM then calculates the required adaptations to the MV settings using the CPM. This illustrates the potential for integrating the deterministic explainability of CPMs and the capacity of AI to handle stochastic data in a combined model.

### Quality standards

A primary aim of this research was to critically appraise the quality of CPMs for individualised MV. An important aspect thereof was the assessment of the adequacy of the validation samples. Validation samples were qualified as ‘satisfactory’ if both the number and type of subjects could be considered to fully represent the adult intensive care population. However, the validation sample was mostly labelled as ‘partly satisfactory’, in case of validation studies based on experimental animals or limited sample sizes. It can be argued that the heterogeneity of the intensive care population requires a sufficiently large amount of granular patient data for representative validation. On the other hand, such ambitious inclusion targets could complicate and limit validation studies, because CPMs often represent complex, difficult to measure physiological phenomena [38]. Highly demanding inclusion targets could be unattainable due to time and budget restraints, which illustrates the need for balanced quality criteria for CPMs in individualised medicine.

The assessment scheme in this review is a conception of quality standards for intensive care medicine based on general guidelines for model verification and validation [12, 39, 40], as specific quality standards for CPMs in the ICU are lacking. Recently, leading regulatory bodies have made considerable efforts to establish an urgently awaited regulatory framework to facilitate the development of CPMs [9, 12, 41]. However, such general regulatory frameworks do not yet constitute standards for CPMs in the ICU. Multidisciplinary expert consensus on such standards for CPMs in intensive care medicine is a fundamental next step in building trust in this technology with clinicians. This requires an open debate among all stakeholders, including, but not limited to, clinicians, technical physicians, nurses, patients, and engineers.

### Model reporting

Another important aspect of the quality assessment was the comprehensiveness of model reporting. None of the articles reported all model quality characteristics to a full extent. Generally, only the most critical assumptions were stated. Although concise quality reporting on successive articles is reasonable for scientific dissemination purposes, it limits a critical appraisal of the model’s appropriate context of use based on the individual articles in this review.

Violation of assumptions may result in inferior model performance. Such violations are more likely to happen when assumptions are reported implicitly, and violations potentially occur unintentionally. As a consequence, erroneous results could ultimately even culminate into suboptimal or even adverse MV management. Likewise, lacking sensitivity and uncertainty analyses limit the user’s ability to appraise the model outcomes, which might result in clinical conclusions that are not valid.

Moreover, the computational models of only ten articles were publicly available. As the mathematical model was reported in almost all articles the computational model can be rebuild in most cases. However, replication of model results is impracticable without the computational model available. The exact computational implementation of the model and how it is adapted to patient data can significantly affect model performance. Therefore, model availability and complete quality reporting are essential for reliable modelling practice, which promote model re-use and transferability to other clinical settings, populations, or applications.

### Non-clinical CPMs

This review focused on physiological respiratory models that are closest to an application in routine clinical care, representing a virtual patient. As a result, 58% of the articles were excluded during full-text screening, as their models were not applicable for individualised MV management in critically ill patients. The models in these articles thus qualify as CPMs of the adult respiratory system, but lack the explicit clinical MV application for individual patients. These CPMs could however offer opportunities in the pre-clinical context and can thereby improve patient care more indirectly. For instance, CPMs have historically proven to be useful to study the mechanisms behind complex pathophysiological concepts as encountered in clinical practice [28, 29], of which the work of D.G. Bates et al. is exemplary, e.g. [42, 43]. These models can also be used to teach pathophysiological concepts in medical training or to optimise clinical protocols by testing devices and alternative treatment strategies in silico.

More recently, the concept of virtual patient trials is emerging to augment real world randomised controlled trials (RCT) [8]. In these so-called in silico trials, the intervention is performed in a digital cohort representative of the real clinical population. Such representativity is more difficult to obtain in real world RCTs, which is related to several factors. On the one hand, RCTs are often performed in highly specific populations to promote study power at the cost of generalisability. On the other hand, the actual study population is subject to chance of the incidental case mix, which could result in reduced specificity by omitting rare but realistic cases. Virtual patient trials could address both these issues by generating large cohorts that are both generalisable and specifically at reduced costs and patient burden.

### Limitations

This systematic literature search focused on articles that explicitly reported the use of CPMs. In case the methodology was not reported as a CPM because of its ubiquity in clinical practice, potentially eligible articles could have been overlooked. Prominent examples of such model-based methods are the commercialised ventilator modes proportional assist ventilation (PAV) (Puritan Bennett™, Minneapolis, MN, USA) and adaptive support ventilation (ASV) (Hamilton Medical AG, Bonaduz, Switzerland). Herein, breathing physiology is expressed as equation of motion, but the computational physiological modelling nature is not explicitly mentioned nor detailed in those publications. Therefore, it is impossible to extract relevant model characteristics and perform adequate quality assessment. We considered it however more important to evaluate the quality of those articles consciously using CPMs, than reviewing the incomplete model reporting in such articles, which would skew the results of the quality assessment. Moreover, as many articles showed to build upon previously published models, the screening of reference lists of eligible studies helped us to identify those articles that were eligible after all.

Additionally, the decision on what qualified as application in individualised MV management was associated with subjective interpretation. This might have affected the inclusion of especially articles with a lower level of readiness, i.e. model proposal, prototyping and development, where clinical utility might be less explicit yet. As such, we might report a slight underrepresentation of CPMs with a low level of readiness. However, including CPMs regardless of clinical applicability would expectedly have yielded an abundance of models with limited relevance to clinical practice, clouding the results on the modelled phenomena and clinical applications.

Lastly, quality assessment is ideally evaluated based on the entire body of work and within a specific intended context of use [39]. However, the modelled phenomena, complexity, study populations and applications evolve dynamically, as illustrated in Fig. 3b, c for the Beacon and CURE Soft models. The successive reporting on different versions of the CPM for various contexts of use, therefore limits appraisal of the developmental process and complicates quality assessment of the model as a whole.

### Future directions

Developing CPMs towards clinical implementation requires a thorough understanding of all levels of clinical readiness and their practical implications for every step in this process [11]. To guide further research and development, a rigorous definition of the intended clinical context of use and adequate data granularity is of pivotal importance at the earliest possible stage of model development (level of readiness 1 and 2). The context of use determines the extend of model complexity and modelled phenomena, setting the stage for future clinical usability at the bedside of an individual patient. Next, as recently endorsed by the FDA, validation, verification, and uncertainty quantification, commonly denominated as VVUQ, should be assessed in a risk-based manner relative to the clinical context of use in the subsequent phases (level 3, 4 and 5) [9]. To this end, the FDA and ASME guidelines are highly recommended [9, 12]. Also the clinical research phases (level 6, 7 and 8) should strongly be guided by the context of use and VVUQ results, as they provide insight into the applicability of the model under specific clinical circumstances.

In this context, it is tempting to speculate whether more complex CPMs like Beacon and CURE Soft will outperform current clinical routines and closed loop ventilation modes like SmartCare/PS and ASV-Intellivent. The ongoing RCTs based on clinical application of these models [13–15] will demonstrate their clinical potential and indicate the most relevant future applications of CPMs, ranging from diagnostics and monitoring to closed-loop control of mechanical ventilators (Fig. 1).

## Conclusion

CPMs for individualised MV management are evolving towards clinical application as explainable virtual patients, culminating in two models being evaluated in RCTs. CPMs are increasingly designed to aid in currently reported main clinical challenges of individualised MV based on mechanistic insights and predominantly dedicated to lung mechanics to optimise lung-protective ventilatory strategies. More recently, CPMs aimed at improved oxygenation and ventilation control are advancing by combining models of gas exchange, gas homeostasis and respiratory control. Recently, also models dedicated to respiratory muscle function for diaphragm-protective ventilation have emerged. Although key models are available for re-use, the model availability is generally limited to the mathematical model and parameters. To promote further development, as well as a widespread and safe clinical application of CPMs in individualised MV, standards for quality assessment and model reporting are pressingly needed.

## Supplementary Information

**Additional file 1:** PRISMA 2020 statement.

**Additional file 2:** Search strategy.

**Additional file 3:** Data extraction form.

**Additional file 4:** Specification of quality assessment criteria in accordance with the ASME V&V40 standard.

**Additional file 5:** List of articles excluded because they did not explicitly state a clinical application in individualised MV management.

**Additional file 6:** Extracted data.

## Data Availability

All data generated or analysed during this study are included in this published article and its supplementary information files.

## Abbreviations

MV: Mechanical ventilation
CPM: Computational physiological model
ASME: American Society of Mechanical Engineers
PEEP: Positive end-expiratory pressure
AI: Artificial intelligence
FDA: Food and Drug Administration
PRISMA: Preferred Reporting Items for Systematic reviews and Meta-analysis
NASA: National Aeronautics and Space Administration
RCT: Randomised controlled trial
PAV: Proportional Assist Ventilation
ASV: Adaptive Support Ventilation
VVUQ: Verification, validation, and uncertainty quantification
