# Supplementary material for: Computational physiological models for individualised mechanical ventilation: a systematic literature review focussing on quality, availability, and clinical readiness
Source: Crit Care. 2023 Jul 6;27:268. doi: 10.1186/s13054-023-04549-9 (PMC10327331; doi:10.1186/s13054-023-04549-9)
Supplement: Supplementary file 2 — Additional file 2: Search strategy. [file 13054_2023_4549_MOESM2_ESM.docx]

**Electronic supplementary materials 2 - Computational physiological models for individualised mechanical ventilation: a systematic literature review focussing on availability, quality and clinical readiness**

R.S.P. Warnaar MSc ^1^, M.P. Mulder MSc ^1^, L. Fresiello PhD ^1^, A.D. Cornet, MD PhD ^2^, L.M.A. Heunks MD PhD ^3^, D.W. Donker MD PhD ^1,4^, E. Oppersma PhD ^1^

^1^ Cardiovascular and Respiratory Physiology, Technical Medical Centre, University of Twente, Enschede, the Netherlands

^2^ Department of Intensive Care, Medisch Spectrum Twente, Enschede, the Netherlands

^3^ Department of Intensive Care, Erasmus University Medical Centre, Rotterdam, The Netherlands

^4^ Intensive Care Centre, University Medical Centre Utrecht, Utrecht, the Netherlands

**Correspondence:**

Rob Warnaar, MSc

Cardiovascular and Respiratory Physiology

Technical Medical Centre, University of Twente

Technohal 3184

P.O. Box 217

7500 AE Enschede

The Netherlands

tel.: +31534899652

mail: r.s.p.warnaar@utwente.nl

# ESM2 Search strategy

## Search query

| DB | Query | #Hits |
| --- | --- | --- |
| PubMed | (  (Comput* OR "in silico" OR virtual OR mathematical OR digital)  AND (respiratory OR ventilatory OR lung OR breath)  AND (Physiology OR physiolog* OR personalis* OR personaliz* OR individualis* OR individualiz* OR patient-specific)  AND (model OR simulat*)  AND (ICU OR intensive care OR critical care OR ventilation)  ) | 3,801 |
| Scopus | TITLE-ABS-KEY (  (comput* OR “in silico” OR virtual OR math* OR digital)  AND (respirat* OR ventilat* OR lung OR breath*)  AND (physiolog* OR personalis* OR personaliz* OR individualis* OR individualiz* OR patient-specific)  AND (model* OR simulat*)  AND (ICU OR (intensive AND care) OR (critical AND care) OR ventilat*)  ) | 3,018 |
| Web of Science | (  ALL=(Comput* OR “in silico” OR virtual OR math* OR digital)  AND ALL=(respirat* OR ventilat* OR lung OR breath*)  AND ALL=(Physiology OR physiolog* OR personalis* OR personaliz* OR individualis* OR individualiz* OR patient-specific)  AND ALL=(model* OR simulat*)  AND ALL=(ICU OR (intensive AND care) OR (critical AND care) OR ventilat*)  ) | 1,824 |
| Embase | (  (comput* OR “in silico” OR virtual OR math* OR digital)  AND (respirat* OR ventilat* OR lung OR breath*)  AND (physiolog* OR personalis* OR personaliz* OR individualis* OR individualiz* OR patient-specific)  AND (model* OR simulat*)  AND (ICU OR (intensive AND care) OR (critical AND care) OR ventilat*)  ) | 4,226 |
