# Supplementary material for: Computational physiological models for individualised mechanical ventilation: a systematic literature review focussing on quality, availability, and clinical readiness
Source: Crit Care. 2023 Jul 6;27:268. doi: 10.1186/s13054-023-04549-9 (PMC10327331; doi:10.1186/s13054-023-04549-9)
Supplement: Supplementary file 3 — Additional file 3: Data extraction form. [file 13054_2023_4549_MOESM3_ESM.pdf]

2. A2 Model family

Is the model based on a previously published model?
Pleasy specify the original article(s).
Mechanical ventilation: Rayyan-ID of full-text screening phase, otherwise author (year).
Multiple original articles can be entered as multiple entries.

If parent model(s), specify:

-- Select --

- ○ Original model
- ○ Parent models

- -- Select or type

3. A1-MV Clinical application

What is the (intended) clinical application?
Timing settings: respiratory rate, cycle-off, trigger threshold, I:E ratio

If

-- Select --

- ☐ FiO2 setting
- ☐ PEEP setting
- ☐ Driving pressure/Tidal volume setting
- ☐ Flow settings
- ☐ Timing settings
- ☐ Pressure monitoring
- ☐ Respiratory drive monitoring
- ☐ Unspecified/Ill-defined/General
- ☐ Other:

- -- Select or type

4. A1b Clinical application type/use

What is the intended way the model supports the clinical process? How can the model be used
Diagnostic: It provides information on the current status of the patient.
Clinical decision support: It advises the clinician what therapy settings to use.
Closed loop control: It adjust therapy settings automatically
Other: ...

If other, specify:

-- Select --

- ☐ Diagnostic
- ☐ Clinical decision support
- ☐ Closed loop control
- ☐ Other
- ☐ General/unknown
- ☐ Pre-clinical testing
- ☐ Mechanistic insight

- -- Select or type

5. B1 Study design

What is the study design/studied context of this article?
In vitro / ex vivo / physical: cells, organs, corpse, mock loop

If other, specify:

- ☐ In silico: only computer simulations
- ☐ In vitro / ex vivo / physical
- ☐ In vivo: lab, animal, healthy human
- ☐ Clinical - Retrospective
- ☐ Clinical - Prospective observational
- ☐ Clinical - Non-randomised clinical trial
- ☐ Clinical - Randomised clinical/controlled trial (RCT)
- ☐ Other:

6. Sample size experimental data: # of subjects

7. B2-MV Modelled physiological phenomena

Lung mechanics: Compliance, resistance, airway/alveolar collapse
Gas homeostasis: Acid base balance, O2 transport
External factors: pharmacological, mechanical support, CPR, gravity, posture, stress, exercise

If other, specify:

- ☐ Lung mechanics
- ☐ Gas exchange/Diffusion
- ☐ Ventilation/Perfusion matching
- ☐ Gas homeostasis
- ☐ Respiratory control/Respiratory drive
- ☐ Respiratory muscle function
- ☐ External factors
- ☐ Other:
- ☐ Unknown

-- Select --

10. B4 Level of validation

What is the level of validation of the model?
None: No validation was performed.
Prior validation: The model was validated in a prior study
General: The model is validated against general physiology.
Patient group: The model is validated to group level characteristics of the patient group of interest.
Individual: The model is validated to personal patient data.

- ○ None
- ○ Prior validation
- ○ General
- ○ Patient group
- ○ Individual

11. B5 Type of validation data

Experimental: animal, healthy human, cells

If other, specify:

- ☐ Literature
- ☐ Experimental
- ☐ Patient
- ☐ Other:

12. B6 Sample size validation data: # of subjects

13. B7 Personalisation: To what extend is the model adapted to the individual patient?

Patient group: The model is adapted to group level characteristics of the patient group of interest.
Individual: The model is adapted to the personal characteristics of a single patient

- ○ Not
- ○ Individual

14. B8 Type of personalisation data

Demographic data: age, weight, length, gender
Routine clinical data: HR, ABP, ECG, RR, temp, lab
Additional measures: PAC, Swan Ganz, echo, CT, MRI
Other: …
Unknown

If other, specify:

- ☐ Demographic data
- ☐ Routine clinical data
- ☐ Additional measures
- ☐ Other:
- ☐ Unknown

-- Select --

- -- Select or type

1. C1 Model level of readiness

C1 Model level of readiness:

- ☐ Problem identification (level 1)
- ☐ Proposal of solution (level 2)
- ☐ Model prototyping (level 3)
- ☐ Model development (level 4)
- ☐ Model validation (level 5)
- ☐ Real-time testing (level 6)
- ☐ Workflow integration (level 7)
- ☐ Clinical testing (level 8)

- [ ] Integration in clinical practice (level 9)

2. C2 Accessibility of computational model

What parts of the model are accessible as indicated by the article?
C1 Model level of readiness:

- [ ] Unknown
- [ ] Mathematical model
- [ ] Used parameters
- [ ] Computational model - Open source
- [ ] Computational model - Freely available application
- [ ] Computational model - Commercialised application

3. C3 Simulation time

Real-time: The model is able to keep track of the patient status in real-time/continuously.
Asynchronous: The model updates/needs to be updated once in a while to keep track of the patient's status.
C1 Model level of readiness:

- ○ Asynchronous: non real-time
- ○ Real-time
- ○ Unknown

4. C4 Time horizon

What is the time horizon of the model: The time range for which the model results are predicted?
C1 Model level of readiness:

Notes

- ○ < Seconds
- ○ Seconds
- ○ Minutes
- ○ Hours
- ○ Days
- ○ > Month
- ○ Unknown

1. QA1a [MODEL RISK] Model influence:

What is the influence of the computational model on decision making:
a. Minor: Other sources of evidence are the main decision determinants
b. Moderate: The model is one of the main decision determinants
c. Significant: The model is the main decision determinant
d. Unknown

- ○ Minor
- ○ Moderate
- ○ Significant
- ○ Unknown

2. QA1b [MODEL RISK] Model consequence

What is the significance of an adverse outcome resulting from an incorrect model output:
a. Low: No adverse effects to patient safety or health, but might result in a nuisance to the physician or might have other impacts.
b. Medium: Minor patient injury or need for physician intervention, or have other moderate impacts.
c. High: Severe patient injury or death, or have other significant impacts.
d. Unknown

- ○ Low
- ○ Medium
- ○ High
- ○ Unknown

3. QA2a [VERIFICATION] To what extent is the numerical code verified?

To what extent is the numerical code verified?
a. None: Numerical Code Verification was not performed.
b. Other code: The numerical solution was compared to an accurate benchmark solution from another verified code.
c. Exact solution: Discretization error was quantified by comparison to an exact solution, and a grid convergence study demonstrated that the numerical solution asymptotically approached the exact solution as the discretization was refined.
d. In addition to the quantification of discretization error and the execution of a grid convergence study as described in (c), the observed order of accuracy was quantified and compared to the theoretical order of accuracy.

Notes

- ○ None
- ○ Other code
- ○ Exact solution
- ○ Exact solution + quantified order of accuracy
- ○ Unknown

4. QA3a [VALIDATION] Computational model: Model form (assumptions)

a. None: Influence of model form assumptions was not explored/described.
b. Partial: Influence of expected key model form assumptions was explored/described.
c. Full: Comprehensive evaluation of model form assumptions was conducted/described.

Notes

- ○ None
- ○ Partial
- ○ Full

5. QA3b [VALIDATION] Computational model: To what extent was sensitivity analysis of model inputs performed?

a. None: Sensitivity analysis was not performed.
b. Partial: Sensitivity analysis on expected key parameters was performed.
c. Full: Comprehensive sensitivity analysis was performed.

- ○ None

- ◯ Partial
- ◯ Full

6. QA3c [VALIDATION] Computational model: Uncertainty quantification of model inputs:

a. None: Uncertainties were not identified.
b. Partial: Uncertainties on expected key inputs were identified and quantified, but were not propagated to quantitatively assess the effect on the simulation results.
c. Full: Uncertainties on all inputs were identified and quantified, and were propagated to quantitatively assess the effect on the simulation results.

- ◯ None
- ◯ Partial
- ◯ Full

7. QA3d [VALIDATION] Comparator: To what extent do the test samples and conditions for validation represent the full context of use?

i.e. to what extent are the characteristics of subjects in the validation sample similar to that of the population in the context of use?
a. Not applicable: No validation was performed.
b. Unsuitable: The test sample and conditions are outside the context of use.
c. Partial: The test sample (type & number) and conditions (type & range) partially represent the context of use.
d. Full: The test sample (type & number) and conditions (type & range) cover the full context of use.
e. Unknown: The test sample and conditions are not or insufficiently described.

Notes

- ◯ Not applicable
- ◯ Unsuitable
- ◯ Partial
- ◯ Full
- ◯ Unknown

8. QA3e [VALIDATION] Assessment: Rigor of output comparison: What method is used to compare to model outputs to the comparator?

a. Not applicable: No validation was performed.
b. Visual: Visual comparison was performed.
c. Arrhythmic difference: Comparison was performed by determining the arithmetic difference between computational results and experimental results.
d. Model uncertainty quantification: Uncertainty in the output of the computational model or the comparator was used in the output comparison.
e. Model and comparator uncertainty quantification: Uncertainties in the output of the computational model and the comparator were used in the output comparison.

- ◯ Not applicable
- ◯ Visual
- ◯ Arrythmic difference
- ◯ Model uncertainty quantification
- ◯ Model and comporator uncertainty quantification

9. QA3f [VALIDATION] Assessment: Agreement of Output Comparison

This component refers to the qualitative or quantitative agreement between the QOIs from the computational model and those from the comparator. NOTE: A satisfactory level of agreement may be assessed based on criteria established for the COU by the practitioner.
a. Not applicable: No validation was performed.
b. Not satisfactory: The level of agreement of the output comparison was not satisfactory for key comparisons.
c. Somewhat satisfactory: The level of agreement of the output comparison was satisfactory for key comparisons, but not all comparisons.
d. Satisfactory: The level of agreement of the output comparison was satisfactory for all comparisons.

- ○ Not applicable
- ○ Not satisfactory
- ○ Somewhat satisfactory
- ○ Satisfactory

10. QA4a [APPLICABILITY] To what extent are the Quantities of Interest (QOIs) of validation related to the QOIs in the Context of Use (COU)?

This comprises the relevance and directness of the validation relative to the COU.
a. Not applicable: No validation was performed.
b. Related: The QOIs from the validation activities were related, though not identical, to those for the COU.
c. Subset identical: A subset of the QOIs from the validation activities were identical to those for the COU.
d. Completely identical: The QOIs from the validation activities were identical to those for the COU.

- ○ Not applicable
- ○ Related
- ○ Subset identical
- ○ Completely identical
