# Supplementary material for: Computational physiological models for individualised mechanical ventilation: a systematic literature review focussing on quality, availability, and clinical readiness
Source: Crit Care. 2023 Jul 6;27:268. doi: 10.1186/s13054-023-04549-9 (PMC10327331; doi:10.1186/s13054-023-04549-9)
Supplement: Supplementary file 4 — Additional file 4: Specification of quality assessment criteria in accordance with the ASME V&V40 standard. [file 13054_2023_4549_MOESM4_ESM.docx]

| **Model characterisation** | | | | |
| --- | --- | --- | --- | --- |
|  | **Full** | **Partial** | **None** | **Unknown** |
| **Verification** | To exact solution | To other code/Model | None | Not described |
| **Assumptions** | Comprehensive evaluation of model form assumptions was conducted/described. | Influence of expected key model form assumptions was explored/described. | Influence of model form assumptions was not explored/described. | *Not applicable* |
| **Sensitivity** | Comprehensive sensitivity analysis was performed. | Sensitivity analysis on expected key parameters was performed. | Sensitivity analysis was not performed. |  |
| **Input data uncertainty** | Uncertainties on all inputs were identified and quantified, and were propagated to quantitatively assess the effect on the simulation results. | Uncertainties on expected key inputs were identified and quantified, but were not propagated to quantitatively assess the effect on the simulation results. | Uncertainties were not identified. |  |
| **Model validation** | | | | |
|  | **Satisfactory** | **Moderately satisfactory** | **Unsatisfactory** | **Not applicable** |
| **Sample** | Full: The test sample (type & number) and conditions (type & range) cover the full context of use. | Partial: The test sample (type & number) and conditions (type & range) partially represent the context of use. | Unsuitable OR Unknown: The test sample and conditions are outside the context of use OR are not or insufficiently described. | No validation was performed. |
| **Rigor of output comparison** | Model AND/OR comparator uncertainty: Uncertainties in the output of the computational model and/or the comparator were used in the output comparison. | Arrhythmic difference: Comparison was performed by determining the arithmetic difference between computational results and experimental results. | Visual: Visual comparison was performed. |  |
| **Agreement of output comparison** | The level of agreement of the output comparison was satisfactory for all comparisons. | The level of agreement of the output comparison was satisfactory for key comparisons, but not all comparisons. | The level of agreement of the output comparison was not satisfactory for key comparisons. |  |

*Adapted from: The American Society of Mechanical Engineers (2018) ASME V&V 40-2018 - Assessing Credibility of Computational Modeling Through Verification and Validation: Application to Medical Devices. New York, NY: The American Society of Mechanical Engineers; 2018. 1–60 p. ISBN: 9780791872048. Available from: https://www.asme.org/codes-standards/find-codes-standards/v-v-40-assessing-credibility-computational-modeling-verification-validation-application-medical-devices.*
